# Biodegradation of real petroleum wastewater by immobilized hyper phenol-tolerant strains of *Bacillus cereus* in a fluidized bed bioreactor

**DOI:** 10.1007/s13205-016-0447-1

**Published:** 2016-06-20

**Authors:** Aditi Banerjee, Aloke K. Ghoshal

**Affiliations:** 1 Centre for the Environment, Indian Institute of Technology Guwahati, Guwahati, Assam 781039 India; 2 Department of Chemical Engineering, Indian Institute of Technology Guwahati, Guwahati, Assam 781039 India

**Keywords:** Fluidized bed bioreactor, Petroleum wastewater, Biodegradation, Immobilized biomass, *Bacilluscereus*

## Abstract

Microbial bioremediation of petroleum wastewater by phenol-degrading-bacteria holds promise in circumventing the issue of petroleum-spill related pollution. Herein, biodegradation of petroleum wastewater samples collected from oil refinery site was carried out in a fluidized bed bioreactor by Ca-alginate immobilized biomass of phenol-degrading strains of *Bacillus cereus* (AKG1 MTCC9817 and AKG2 MTCC9818). Degradation performance of the system was evaluated by measuring the changes in chemical oxygen demand (COD) and level of phenolic compounds in the wastewater samples during the microbial treatment. The microbial treatment reduced initial COD level and concentration of phenolic compounds by 95 % or more, demonstrating the excellent efficacy of immobilized AKG1 and AKG2 strains in treating petroleum wastewater in continuous mode of operation. The present study demonstrates the potential of immobilized AKG1 and AKG2 in treating petroleum wastewater in fluidized bed bioreactors.

## Introduction

Various types of bioreactors have shown their potential for microbial degradation of toxic pollutants, especially in continuous mode of operation. Among these bioreactors, fluidized bed bioreactor (FBR) is one of the best and present many advantages related to hydrodynamics and mass transfer phenomena. Use of fluidization as a contact technique has gained considerable importance in the biochemical field (Schügerl 1997) as FBR gives intimate contact between the liquid and solid phases (Shieh and Keenan 1986; Worden and Donaldson 1987). Biological treatment of industrial wastewaters, in general, requires a great deal of space as well as time (retention time can be many days) when using activated sludge or aerated lagoon -based systems (Hüppe et al. 1990; Shieh and Keenan 1986; Wu and Wisecarver 1992). An FBR, on the other hand, is capable of achieving treatment in low retention time because of the high biomass concentration that can be attained in a bioreactor (Shieh and Keenan 1986; Yoong et al. 1997). While similar to a trickling filter (a packed-bed fixed-film bioreactor) in treatment concept, FBR offers distinct mechanical advantages which allow small and high surface area material to be used for biomass growth (Shieh and Keenan 1986). Fluidization overcomes operating problems, such as bed clogging and high pressure drop, which would occur if small materials with high surface area were employed in packed-bed operation (Fernández et al. 2008). Rather than clogging with new biomass growth, the FBR bed simply expands. Thus, for comparable treatment efficiency, the required bioreactor volume is greatly reduced (Shieh and Keenan 1986). Hirata et al. (2000) and Safferman and Bishop (1996) reported that FBR has active transfer phenomena accelerated by fluidizing materials, and the reactor retains 5–10 times higher biomass concentration than conventional activated sludge reactors. Subsequently, much attention has been directed to FBRs recently—over other suspended-growth systems—due to high biomass concentrations, low hydraulic residence times, small footprint requirements, and absence of mechanical moving parts (Lohi et al. 2008) in FBRs.

Reports indicate that oil will remain a major source of energy for several decades to come. Thus, the pollution as result of oil spillage during manufacturing and transportation of refinery products poses a major challenge. With respect to continuous microbial treatment of petroleum wastewater containing several toxic organic compounds, Karamanev and Margaritis (2005) reported the biodegradation of petroleum hydrocarbons in an immobilized cell airlift bioreactor using soil contaminated with petroleum hydrocarbons as the source of naturally occurring microorganisms. Lohi et al. (2008) treated diesel fuel-contaminated wastewater aerobically in a three-phase fluidized bed reactor under the unsteady- and steady-state conditions. Attached biomass, in this regard, has been claimed to be more resistant toward toxicity than freely suspended ones (Pedersen and Arvin 1995). Immobilized biomass is preferred to free cells in FBRs as it offers several advantages, such as reduced mechanical stress, elimination of secondary clarifier, and ease of sludge disposal (Fernández et al. 2008).

With the aim of microbial treatment of petroleum wastewater, we have recently isolated two strains of *B.* *cereus* (AKG1 MTCC9817 and AKG2 MTCC9818) which show high phenol-tolerance as well as phenol-degradability (Banerjee and Ghoshal 2010a, b). Evaluation of the biodegradation performance of these isolated strains in a FBR, in this regard, will enrich our knowledge to assess the feasibility of using AKG1 and AKG2 in practical bioremediation of petroleum wastewater. In the present study, microbial treatment of real petroleum wastewater by the immobilized strains of isolated *B. cereus* AKG1 MTCC9817 and *B. cereus* AKG2 MTCC9818 has been carried out in a recirculated up-flow FBR to accumulate preliminary information about the degradation behavior of the strains in the FBR.

## Materials and methods

### Growth of bacteria and immobilization of bacterial cells in alginate beads

The bacterial strains *B. cereus* AKG1 MTCC9817 and *B. cereus* AKG2 MTCC9818, isolated from wastewater samples as reported previously (Banerjee and Ghoshal 2010a, b, 2011), were grown and maintained in phenol-containing mineral salt medium (MSM: 4.0 g L^−1^ sodium nitrate (NaNO_3_), 3.61 g L^−1^ disodium hydrogen phosphate (Na_2_HPO_4_), 1.75 g L^−1^ potassium dihydrogen phosphate (KH_2_PO_4_), 0.2 g L^−1^ magnesium sulfate (MgSO_4_·7H_2_O), 0.05 g L^−1^ calcium chloride (CaCl_2_·2H_2_O), 1.0 mg L^−1^ ferrous sulfate (FeSO_4_·5H_2_O), 50 μg L^−1^ copper sulfate (CuSO_4_·5H_2_O), 10 μg L^−1^ sodium molybdate (Na_2_MoO_3_), 10 μg L^−1^ manganese sulfate (MnSO_4_), and 3 g L^−1^ Yeast extract). The bacteria were grown at 37 °C with constant shaking (120 rpm) in culture media with pH adjusted to 7.0 and 7.5 for the strains AKG1 and AKG2, respectively (optimum growth conditions for the strains).

Following bacterial growth for 12 h, bacterial cells were collected by centrifugation (5000 rpm, 10 min). The bacterial cells (AKG1 and AKG2) were resuspended together in phosphate buffered saline (PBS) and mixed with sterilized alginate solution (3 %, w/v, 200 mL) on a magnetic stirrer. When the resulting mixture was extruded drop by drop into cold calcium chloride solution (CaCl_2_, 0.1 M), alginate-cell solution was gelled forming uniform-sized (~0.35 cm) Ca-alginate beads with entrapped bacterial cells. After being aged in CaCl_2_ (0.2 M) solution for 1 h, the beads were rinsed with MSM and distilled water to remove any excess CaCl_2_.

### Setup of fluidized bed reactor for continuous biodegradation

**Fig. 1:**
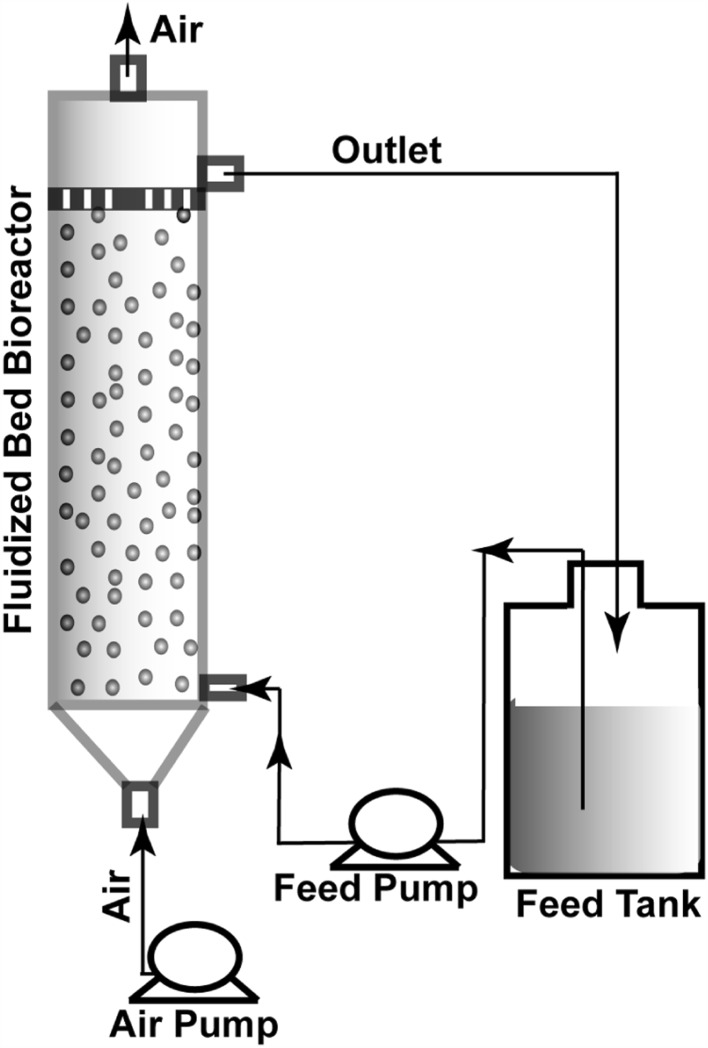
Schematic representation of the recirculated up-flow fluidized bed bioreactor (dimensions are not in* scale*)

The FBR used for the biodegradation of petroleum wastewater in the present study was a tubular system with an internal diameter of 5 and 100 cm in height, as shown schematically in Fig. 1. The active bed volume (55 cm^3^) was packed with immobilized alginate beads (diameter = 0.3 cm; containing AKG1 and AKG2 equivalent to 16.87 g of dried microorganism). The average density of immobilized beads was 1.254 g cm^−3^. Pre-autoclaved refinery wastewater (1.2 L, collected from local refinery site) was used as feed to the reactors from the bottom in an up-flow mode of operation. The flow rate of the feed was kept constant at 6 mL min^−1^, and an air-flow of 1 LPH was maintained during the entire period of experiment. Total feed was fluidized by air circulation. The minimum air fluidization velocity was experimentally measured to be 0.306 LPH, and elution velocity was 52.8 LPH. All the biodegradation experiments were carried out at room temperature of 30 °C. Feed samples were collected at regular intervals from the top of the reactor for further analysis.

### Analysis of chemical oxygen demand (COD) and phenolic compounds

The chemical oxygen demand (COD) of the sample was estimated by the dichromate method (closed reflux, titrimetric method) (APHA 2005). For this, properly diluted samples were added to the digestion solution (0.01667 M potassium dichromate), followed by the addition of sulfuric acid reagent and digestion at 150 °C for 2 h. The digested mixture, cooled to room temperature and mixed with Ferroin indicator (0.05 mL), was titrated with ferrous ammonium sulfate (FAS, 0.01 M). The end point was a change of color from blue-green to reddish brown. The COD value was calculated as mg O_2_ L^−1^ using the equation, $\text{COD} = \frac{(A - B) \times M \times 8000}{{\text{mL} \, \text{sample}}}$, where, *A* is the FAS used for blank (mL), *B* the FAS used for sample (mL), *M* the molarity of FAS, and the factor 8000 is the milli-equivalent weight of oxygen ×1000 mL L^−1^.

The concentration of phenolic compounds was estimated by spectrophotometric method (APHA 2005), where 2.5 mL NH_4_OH (0.5 N) solution was mixed to 100 mL of diluted sample and the pH maintained to 7.9. After that, 1 mL 4-aminoantipyrine and 1 mL potassium ferricyanide solution were added in sequence and mixed well. Following 15 min incubation, absorbance of the colored dye was measured at 500 nm, and concentration of phenolics was calculated from the calibration curve constructed from the phenol standards.

## Results and discussion

**Fig. 2:**
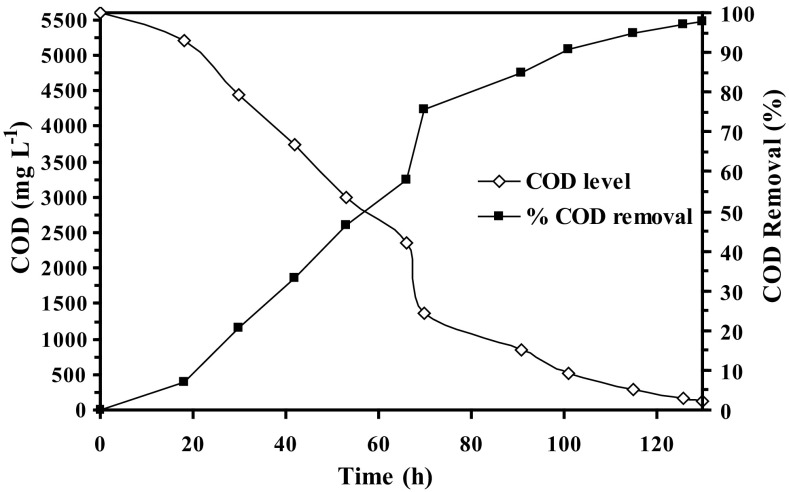
The COD level and the corresponding COD removal (%) in the refinery wastewater during the microbial treatment in fluidized bed reactors by Ca-alginate immobilized AKG1 and AKG2

COD is often used as an indicator of water quality as it measures the amount of organic compounds present in wastewater. Initial COD level of the refinery wastewater in the present study, prior to the microbial treatment, was measured to be 5600 mg L^−1^. The percentage COD removal from refinery wastewater in the FBR by the *B. cereus* (AKG1 and AKG2) immobilized in Ca-alginate beads is shown in Fig. 2. From Fig. 2, it is evident that the COD removal by the immobilized bacterial strains gradually increased as the microbial treatment progressed. For 50 % COD removal, it took around 53 h. Furthermore, on the completion of the biodegradation experiment (~130 h), COD level of the wastewater was reduced by 97.86 %. After 130 h of microbial treatment, final COD level of the refinery wastewater reached a value of 50 mg L^−1^. The results essentially demonstrate that the high level of COD (5600 mg L^−1^) in the refinery wastewater was successfully removed by immobilized AKG1 and AKG2 in the FBR.

**Fig. 3:**
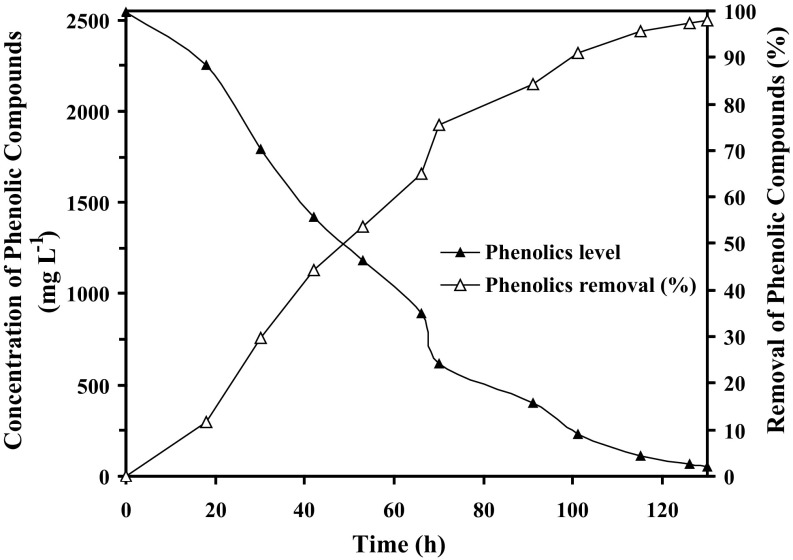
Concentration of phenolic compounds and the corresponding removal (%) in the refinery wastewater during the microbial treatment in fluidized bed reactors by Ca-alginate immobilized AKG1 and AKG2

Phenol and other phenol derivatives are the major organic compounds present in refinery wastewater and have a direct relation with COD (1 mg phenol ≡ 2.38 mg COD) (Tyagi et al. 1993). The initial concentration of phenolic compounds in the petroleum wastewater at the beginning of the microbial treatment was measured to be as high as 2545 mg L^−1^. Figure 3 shows the level of phenolic compounds in the petroleum wastewater and subsequent removal (due to biodegradation) during the microbial treatment in the fluidized bed bioreactor by immobilized bacterial strains. In the FBR, the phenolic compounds were biodegraded almost linearly in the initial period of the microbial treatment up to 70 h (~3 days) resulting in 75.63 % removal of the phenolics present in the refinery wastewater. Subsequently, the rate of phenolics removal decreased, and the phenolic compounds of the wastewater were completely (98.03 %) removed after 130 h (~6 days) of treatment. As evident from Fig. 3, removal of phenolic compounds (%) by the immobilized systems followed the same trend as that of COD removal (Fig. 2) demonstrating the relation of phenolic compounds with COD as discussed earlier. It may be mentioned here that the identification of products of phenol biodegradation by immobilized AKG1 and AKG2 is important and warrants future investigation.

In conclusion, present investigation on continuous biodegradation of the petroleum refinery wastewater in recirculated FBR by the isolated strains of AKG1 and AKG2 immobilized in Ca-alginate beads demonstrated that the microbial treatment reduced initial COD level and concentration of phenolic compounds by 95 % or more, indicating excellent efficacy of the immobilized AKG1 and AKG2 strains in treating petroleum wastewater in continuous mode of operation. Overall, our study strongly suggests the potential of immobilized AKG1 and AKG2 in the treatment of petroleum wastewater in FBRs and provides preliminary results in this regard.
